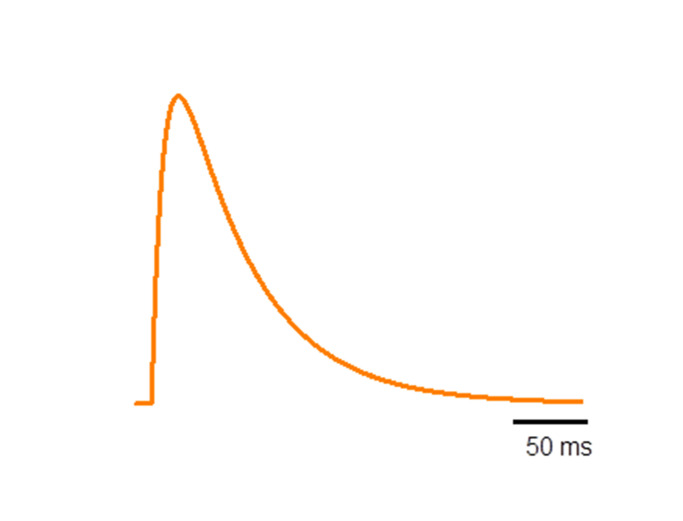# Correction: Thermal modulation of epicardial Ca^2+^ dynamics uncovers molecular mechanisms of Ca^2+^ alternans

**DOI:** 10.1085/jgp.20201256803092021c

**Published:** 2021-03-26

**Authors:** Jose Millet, Yuriana Aguilar-Sanchez, Dmytro Kornyeyev, Maedeh Bazmi, Diego Fainstein, Julio A. Copello, Ariel L. Escobar

Vol. 153, No. 2 | 10.1085/jgp.202012568 | January 07, 2021

During production of this article, we inadvertently omitted an image from the Supplemental text. The beginning of the Supplemental text should have appeared as below. The error only exists in PDFs downloaded before March 9, 2021.

## Supplemental material

Let’s have a function *f*(*t*) that describes the kinetics of a Ca^2+^ transient:

$$f\left(t\right)=\left(1-e^{-\frac{t}{\tau_{οn}}}\right)•e^{-\frac{t}{\tau_{\mathrm{off}}}}\cdot$$

**Figure 12:**